# Genetically Predicted Gut Microbiota and Host Metabolites Mediate the Causal Link Between Dietary Factors and Acute Myeloid Leukemia

**DOI:** 10.1002/fsn3.70456

**Published:** 2025-06-23

**Authors:** Jiading Qin, Ling Zhang, Guangcheng Zhang, Weijie Liao, Li Yu

**Affiliations:** ^1^ Department of Hematology and Oncology, Shenzhen University General Hospital, International Cancer Center, Hematology Institution, Haoshi Cell Therapy Institute of Shenzhen University, Shenzhen University Medical School Shenzhen University Shenzhen China; ^2^ Guangdong Key Laboratory for Biomedical Measurements and Ultrasound Imaging, National‐Regional Key Technology Engineering Laboratory for Medical Ultrasound, School of Biomedical Engineering Shenzhen University Medical School Shenzhen China; ^3^ Jiangxi Provincial Key Laboratory of Hematological Diseases, Department of Hematology, The 2nd Affiliated Hospital, Jiangxi Medical College Nanchang University Nanchang China

**Keywords:** acute myeloid leukemia, gut microbiota, host metabolites, mendelian randomization study, processed meat intake

## Abstract

Dietary factors may have a causative influence on acute myeloid leukemia (AML), a hematologic cancer known to be highly fatal. It is still mostly unknown whether causal mechanisms connect dietary exposures to AML, particularly through the gut microbiota and host metabolism. A two‐sample Mendelian randomization (MR) analysis was conducted using genome‐wide association study (GWAS) data to assess the causal effect of 20 routine dietary factors on AML. A two‐step MR framework was employed to assess the mediating roles of gut microbiota and host metabolites. Sensitivity analyses, including inverse variance weighted (IVW), MR‐Egger, and MR‐PRESSO, were performed to ensure the robustness of results. Processed meat consumption was causally associated with an increased risk of AML (IVW OR = 15.111; 95% CI = 1.253–182.262; *p* = 0.033). The gut microbiota‐associated L‐histidine degradation I pathway was identified as a pro‐carcinogenic mediator, accounting for 19.5% of the effect. Conversely, host metabolites, including circulating glycerol levels and the free cholesterol‐to‐total lipids ratio in very large high‐density lipoprotein (HDL), functioned as protective mediators. No substantial horizontal pleiotropy or heterogeneity was observed, thereby reinforcing the validity of these findings. This research offers a new understanding of the role of processed meat in the development of AML via the microbiota‐metabolite axis and emphasizes possible metabolic compensatory pathways.

## Graphical abstract

Gut microbial and metabolite pathways mediate the dietary influence on AML risk. Processed meat intake was associated with increased AML via L‐histidine degradation. Conversely, higher glycerol and a greater free cholesterol‐to‐total lipid ratio in large HDL correlated with reduced AML risk.

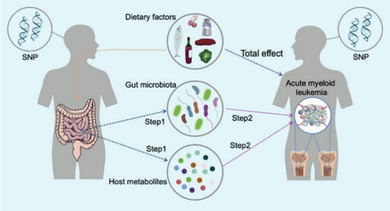

**Abbreviations**
2‐HG: 2‐hydroxyglutarate
AML: acute myeloid leukemia
CE: cholesteryl ester
CIs: confidence Intervals
FC: free cholesterol
FLT3‐ITD: FMS‐like tyrosine kinase 3
GWAS: genome‐wide association studies
HAAs: heterocyclic aromatic amines
HDL: high‐density lipoprotein
ICD‐10: International Classification of Diseases, 10th Revision
IDH: isocitrate dehydrogenase
IMCs: immature myeloid cells
IVs: instrumental variables
IVW: inverse variance weighted
MDS: myelodysplastic syndrome
MR: mendelian randomization
MR‐Egger: mendelian randomization‐egger
MR‐PRESSO: mendelian randomization pleiotropy residual sum and outlier
NDMA: N‐nitrosodimethylamine
NMR: nuclear magnetic resonance
NOCs: N‐nitroso compounds
OR: odds ratio
RCT: randomized controlled trial
SD: standard deviation
SNPs: single‐nucleotide polymorphisms
STROBE‐MR: strengthening the reporting of observational studies in epidemiology using mendelian randomization
TCA cycle: tricarboxylic acid cycle

## 1 Introduction

Acute myeloid leukemia (AML) is a severe hematological malignancy originating from the myeloid cell line, often resulting in bone marrow failure, severe infections, and bleeding. In 2024, approximately 20,800 new cases and 11,220 deaths are expected in the United States (DiNardo et al. 2023; Siegel et al. 2024). Due to the high relapse rate and refractory nature of AML, the five‐year survival rate remains as low as 31.9% (Thol et al. 2024). Beyond its high mortality rate, AML is increasingly acknowledged as a malignancy potentially influenced by modifiable lifestyle factors (Zhou et al. 2024).

Epidemiological evidence suggests that various behavioral patterns, including obesity, smoking, and occupational exposure to benzene and formaldehyde, contribute to the development of hematologic malignancies, including AML (Larsson and Burgess 2022; Poynter et al. 2016; Zhang et al. 2023). For instance, the carcinogenic effects of smoking have been investigated in a genetically homogeneous cohort of AML patients carrying the Fms‐like tyrosine kinase 3 (FLT3‐ITD) mutation (Figueroa et al. 2023). Conversely, moderate‐to‐vigorous physical activity has been identified as a potential protective factor against myeloid malignancies, including AML (Rees‐Punia et al. 2019). Despite these observations, significant gaps remain in our understanding of how lifestyle factors, especially dietary habits, influence AML development (Rezae et al. 2024). Considering the high aggressiveness and poor clinical outcome of AML, dietary interventions may serve as a preventive measure to mitigate disease onset. Elucidating the causal mechanisms of dietary influence on AML is essential for establishing preventative interventions and identifying early diagnostic biomarkers.

Dietary patterns refer to the overall composition and frequency of food intake. With growing public awareness of nutrition and health, an increasing number of diseases have been linked to dietary habits (Collaborators 2019). Accumulating evidence supports causal links between specific dietary factors and cancer development. For example, green tea intake has shown an inverse association with the incidence of gastric cancer and head and neck cancer, while excessive consumption of processed meats has been positively linked to increased risks of breast, colorectal, and lung cancers (Filippini et al. 2020; Huang et al. 2017; Zhang et al. 2024). Recent studies have indicated a potential association between dietary factors and AML, highlighting the possibility that dietary interventions may serve as a promising therapeutic strategy for AML patients (Cunningham et al. 2022). To date, the relationship between dietary factors and AML remains poorly characterized, and research investigating the underlying mechanisms is still markedly limited (Rezae et al. 2024).

The composition and functionality of gut microbiota are profoundly shaped by dietary intake, influencing the balance between immune homeostasis and disease susceptibility (Zmora et al. 2019). Compared to healthy controls, AML patients exhibited an increased abundance of *Streptococcus* and a reduced abundance of *Megamonas* and *Prevotella* (Yu et al. 2021). Emerging evidence indicates that preexisting gut microbiota dysbiosis in leukemia patients triggers pro‐inflammatory immune signaling, which in turn may promote the development and progression of leukemia (Furci et al. 2023). Specifically, AML progression may be driven by gut‐derived mechanisms including epithelial barrier disruption (leaky gut), altered microbial composition, and stimulation of immune pathways via microbe‐associated molecular patterns (ElMokh et al. 2022; Hueso et al. 2020; Yu et al. 2021). These findings underscore the potential of gut microbiota as both a biomarker for early diagnosis of AML and a therapeutic target for disease intervention. In parallel, dietary factors can directly influence host metabolite profiles, which play a pivotal role in regulating tumor initiation and progression (Lien et al. 2021). Aberrant host metabolites play a pivotal role in the pathogenesis of AML, although the precise mechanisms underlying these associations have yet to be fully elucidated. Very‐long‐chain fatty acid metabolism plays a critical role in AML by supporting mitochondrial function and facilitating leukemic cell proliferation (Tcheng et al. 2021). While fatty acid metabolism is essential for leukemic cell survival, its dysregulation can result in elevated lipid peroxidation and oxidative stress, which may ultimately induce ferroptosis in AML cells (Wang et al. 2022). To date, few studies have investigated how microbial communities and host metabolites jointly mediate the effects of dietary factors on AML, thereby limiting our current mechanistic understanding of diet–microbiota–host interactions in AML.

Mendelian randomization (MR) is a robust analytical approach that leverages genetic variants as instrumental variables to overcome key limitations of traditional observational and epidemiological studies, particularly confounding and reverse causation (Sekula et al. 2016). During meiosis, alleles are randomly allocated to offspring in a manner analogous to randomized controlled trials (RCTs) (Emdin et al. 2017). This biological mechanism is typically unaffected by external influences, thereby minimizing both confounding factors and weak instrument bias (Burgess and Thompson 2011). Two‐sample MR further strengthens the validity of findings by measuring exposures and outcomes in separate cohorts, enabling more comprehensive and rigorous evaluation (Davies et al. 2018). By integrating population‐level genetic variants as instrumental variables (IVs), MR enables the estimation of causal effects between exposures and outcomes. Building on existing evidence linking dietary factors, the gut microbiome, metabolites, and AML, this study integrates the latest summary‐level cohort data from genome‐wide association studies (GWAS). Using a two‐sample MR approach, we aim to infer potential causal relationships among these variables. In addition, a two‐step MR approach is applied to examine whether the gut microbiome and host metabolites mediate the causal effect of dietary exposures on AML, thereby offering novel insights for early clinical diagnosis and preventive interventions.

## 2 Materials and Methods

### 2.1 Study Design

**FIGURE 1:**
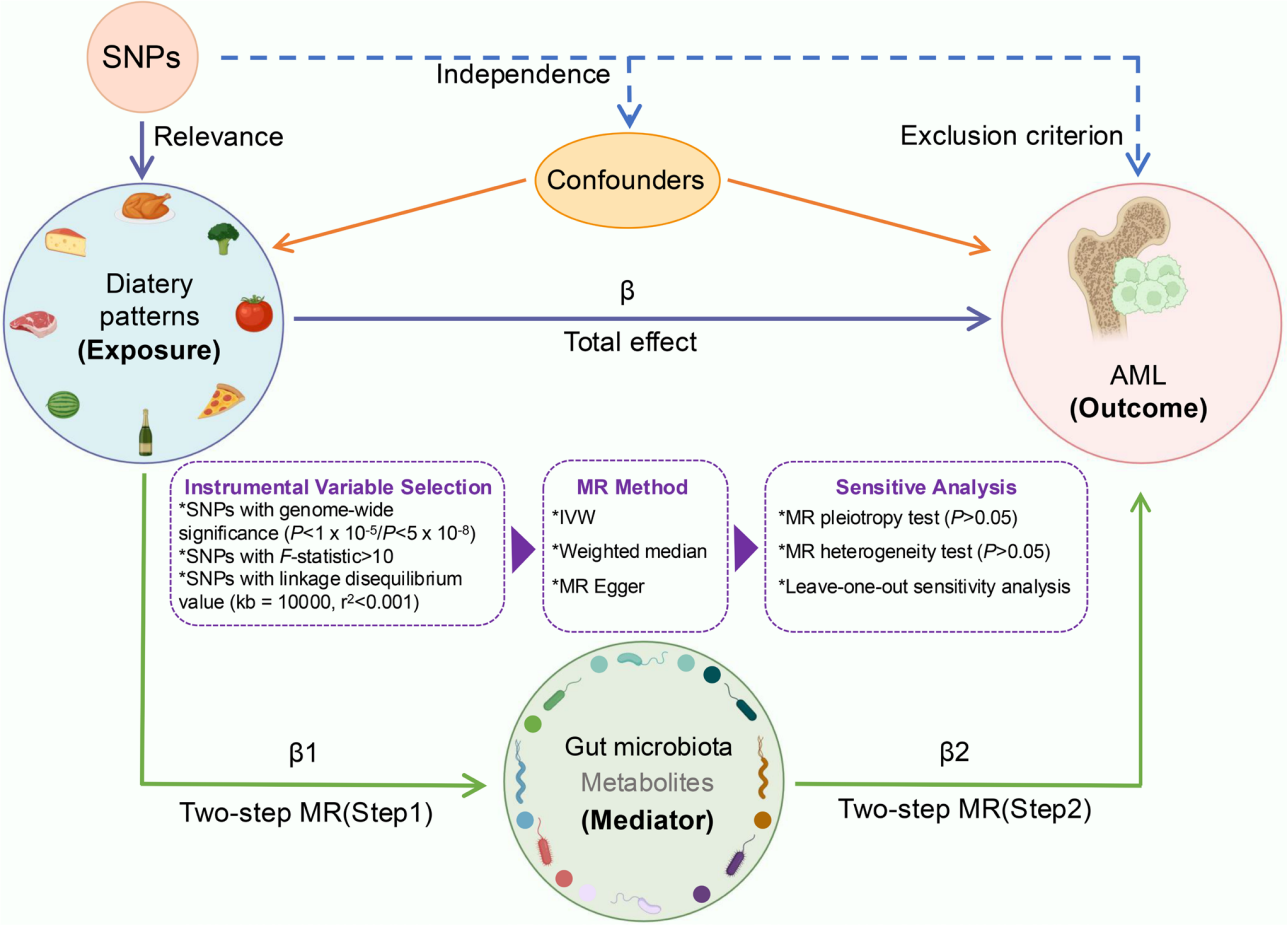
Conceptual framework and key assumptions of this study. The MR approach is based on three fundamental assumptions: (1) instrumental variables (IVs) must exhibit a strong correlation with the exposure of interest (Relevance), (2) IVs should remain unaffected by confounding variables (Independence), and (3) IVs should exert their impact on the outcome exclusively through their association with the exposure, without direct influence (Exclusion Restriction).

Figure 1 illustrates the overall study design. We conducted an MR study using genetic instruments, specifically single‐nucleotide polymorphisms (SNPs), to estimate the causal relationships between exposures and outcomes. Three core assumptions must be satisfied for valid MR analyses: (1) relevance: the genetic instruments should be strongly associated with the exposure, (2) independence: the genetic instruments must be independent of confounding factors, and (3) exclusion restriction: the genetic instruments influence the outcome exclusively through the exposure. Moreover, we employed a two‐step MR framework based on three key conditions: (1) the exposure has a causal effect on the outcome, (2) the mediator has a causal effect on the outcome that is independent of the exposure, and (3) the exposure causally affects the mediator. This study adhered to the STROBE‐MR guidelines (Skrivankova et al. 2021), with detailed items provided in Table S1.

### 2.2 Data Sources

We explored five broad dietary components—meat, vegetable, fruit, drink, and seasoning—that collectively included 20 routine dietary factors (including cheese intake, bread intake, dried fruit intake, salad/raw vegetable intake, beef intake, fish intake, tea intake, pork intake, etc.), as we summarized in Table 1. The GWAS summary statistics for these dietary exposures were obtained from the UK Biobank courtesy of the IEU Open GWAS project (https://gwas.mrcieu.ac.uk/). As we presented in Table S2, these data were collected using standardized protocols at the UKB Assessment Center, where participants completed questionnaires on consumption frequency, enabling estimation of daily dietary intake. As an open‐access resource for public health research, the UK Biobank is one of the largest population‐based biorepositories worldwide, encompassing over 500,000 individuals aged 40–69 (Conroy et al. 2019).

**TABLE 1:** Comprehensive overview of GWAS datasets in this study.

Category	Trait	Ancestry	Unit^a^	Sample size	Consortium/Authors	PMID
Exposure	Cheese intake	European	SD	451,486	UK biobank	NA
	Bread intake	European	SD	452,236	UK biobank	NA
	Dried fruit intake	European	SD	421,764	UK biobank	NA
	Salad/raw vegetable intake	European	SD	435,435	UK biobank	NA
	Beef intake	European	SD	461,053	UK biobank	NA
	Oily fish intake	European	SD	460,443	UK biobank	NA
	Tea intake	European	SD	447,485	UK biobank	NA
	Processed meat intake	European	SD	461,981	UK biobank	NA
	Coffee intake	European	SD	428,860	UK biobank	NA
	Lamb/mutton intake	European	SD	460,006	UK biobank	NA
	Water intake	European	SD	427,588	UK biobank	NA
	Cereal intake	European	SD	441,640	UK biobank	NA
	Hot drink temperature	European	SD	457,873	UK biobank	NA
	Shellfish intake	European	SD	64,939	UK biobank	NA
	Fresh fruit intake	European	SD	446,462	UK biobank	NA
	Salt added to food	European	SD	462,630	UK biobank	NA
	Nonoily fish intake	European	SD	460,880	UK biobank	NA
	Pork intake	European	SD	460,162	UK biobank	NA
	Green tea intake	European	SD	64,949	UK biobank	NA
	Poultry intake	European	SD	461,900	UK biobank	NA
Mediator	Gut microbiome	European	SD	7738	Lopera‐Maya et al.	35115690
	Circulating metabolites	European	SD	120,241	Karjalainen et al.	38448586
Outcome	AML	European	Binary	379,069	FinnGen biobank	36653562

^a^ The unit has two types: SD (Standard Deviation), which represents the variation or spread of a given trait, while Binary refers to an outcome variable with two possible values.

Gut microbiota data were retrieved from the Dutch Microbiome Project, comprising 207 microbial taxa (including 5 phyla, 10 classes, 13 orders, 26 families, 48 genera, and 105 species) and 205 enriched functional pathways (Lopera‐Maya et al. 2022). Taxonomic classifications (species, genus, family, order, class, phylum, kingdom) are annotated using “s_”, “g_”, “f_”, “o_”, “c_”, “p_”, and “k_”, respectively. Subsequently, we included the latest host metabolite data on 233 circulating human metabolic biomarkers quantified by nuclear magnetic resonance (NMR) spectroscopy in up to 120,241 European participants from multiple cohorts (Karjalainen et al. 2024). The outcome data for AML were sourced from the latest FinnGen dataset, which comprises extensive population‐based cohorts in Finland, offering insights from a well‐phenotyped and isolated population (Kurki et al. 2023). In this study, we included 379,069 individuals, including 322 AML cases and 378,747 control participants, with the controls specifically excluding all forms of cancer to ensure the integrity of comparisons. AML patients were diagnosed and classified using ICD‐10 codes, thereby ensuring consistent and reliable identification within the dataset.

To minimize potential population bias, we ensured that all GWAS summary statistics were derived from cohorts of European ancestry, including the UK Biobank, Dutch Microbiome Project, and FinnGen. This ancestry‐matching strategy has been recommended for two‐sample MR analyses to reduce population stratification confounding (Dai et al. 2023; Ge et al. 2025; Yao et al. 2025). All datasets employed standardized phenotyping and genotyping procedures. As our analyses exclusively rely on publicly available summary‐level data, no additional ethical approval was required.

### 2.3 Selection of Genetic Instruments

We applied a rigorous procedure to ensure valid and strong genetic instruments. First, to be considered as IVs, specific SNPs had to be significantly associated with the exposure of interest. For most exposures, we used a genome‐wide significance threshold of *p* < 5 × 10^−8^. However, due to the limited number of genome‐wide significant SNPs for gut microbiota traits, we applied a more lenient threshold of *p* < 1 × 10^−5^, as adopted in previous studies (Ma et al. 2024; Mao et al. 2023; Sincan et al. 2023). Second, we applied a clumping process (window size = 10,000 kb, *r* ^2^ = 0.001) to exclude SNPs in linkage disequilibrium so that the retained SNPs would be independent instruments (Sekula et al. 2016). We aligned the effect alleles between exposure and outcome datasets and removed palindromic SNPs (e.g., with A/T or G/C alleles) and ambiguous or duplicated variants (Gibson et al. 2023). All alleles were standardized to the GRCh37 reference genome. Third, we determined each SNP's instrumental strength by computing the *F*‐statistic, which is derived from both the magnitude and precision of the SNP's genetic influence on the trait. We calculated *F* = *R* ^2^ (*N* − 2)/(1 − *R* ^2^), where (*R* ^2^) is the fraction of phenotypic variance explained by the SNP, and *N* is the GWAS sample size for that trait as previously described (Qin et al. 2023). Moreover, the proportion of variance explained (*R* ^2^) using the formula *R* ^2^ = 2 × EAF × (1‐EAF) × *β* ^2^, where EAF denotes the effect allele frequency of the SNP, and *β* represents its estimated effect on the trait (Hill and Mackay 2004). To avoid weak instrument bias, we excluded any SNP with an *F*‐statistic < 10.

### 2.4 Estimation of Total Causal Effects Predicted by Genetic Instruments

We conducted a two‐sample MR analysis to estimate the total causal effect (*β*) of each dietary factor on AML. The primary analytical approach was inverse variance weighted (IVW) under a fixed‐effects model; if heterogeneity was detected among genetic instruments, we shifted to a random‐effects model (Lin et al. 2021). Notably, IVW assumes that all IVs are valid instruments and estimates the causal effect via the weighted slope in a linear regression framework, producing a robust effect estimate (Burgess et al. 2013). To further assess the consistency of the findings, we also performed the weighted median and MR‐Egger regression methods. The weighted median approach can yield consistent estimates even if up to 50% of the genetic variants are invalid (Bowden et al. 2016). MR‐Egger allows the detection of violations in the standard IV assumptions and provides a pleiotropy‐adjusted causal estimate (Bowden et al. 2015).

### 2.5 Mediation Effect Analysis of Carcinogenetic Effect and Compensatory Effect

We used a two‐step MR approach to dissect both direct and indirect mediated pathways through which dietary factors might affect AML risk via gut microbiota and host metabolites. In the first step, we estimated the effect of each dietary factor on the potential mediator (*β*_1_), and we denoted the overall effect of dietary factors on AML as *β*_total_. In the second step, we investigated whether and how the mediator influenced AML (*β*_2_) (Burgess et al. 2015; Relton and Davey Smith 2012). The percentage of mediation was calculated using the product of coefficients method, where the mediated effect was defined as *β*_1_ × *β*_2_, and the proportion mediated was computed as (*β*_1_ × *β*_2_)/*β*_total_, following the approach described in the previous study (Rogne et al. 2024). Notably, the SNPs utilized in the second step were independent of those employed in the first step, ensuring no overlap of genetic instruments. Considering both the carcinogenetic effect and the compensatory effect among the gut microbiota and host metabolites, we calculated their mediation, respectively.

### 2.6 Sensitivity Analysis

We performed multiple sensitivity analyses to test the robustness of our results regarding potential heterogeneity, pleiotropy, and the influence of individual SNPs. The procedures are as follows: First, horizontal pleiotropy can lead to spurious associations when genetic instruments affect other traits related to the outcome. We used MR‐Egger regression to detect horizontal pleiotropy by examining the intercept term (Burgess and Thompson 2017). A significant intercept suggests the existence of pleiotropy (*p* < 0.05). Second, in the presence of pleiotropy, we employed the MR‐PRESSO method to detect and correct for bias due to outlier SNPs (Verbanck et al. 2018). If MR‐PRESSO indicated the presence of pleiotropy, we deemed the results unreliable and excluded them from subsequent two‐step MR analyses. Next, we used Cochran's Q test to evaluate heterogeneity among IVs (Cohen et al. 2015). *p* < 0.05 suggests significant heterogeneity. If heterogeneity was detected, we adopted the IVW random‐effects model. Finally, our study conducted leave‐one‐out analysis, sequentially removing each SNP to evaluate whether any single variant excessively influenced the overall estimate (Burgess and Thompson 2011). Substantial change in the results upon removal of a specific SNP would warrant further scrutiny.

### 2.7 Data Analyses

All MR analyses and causal effect estimations were performed in R (version 4.4.1) using the “TwoSampleMR” (version 0.6.8) package. The MR‐PRESSO test was conducted using the “MR‐PRESSO” (version 1.0) package. We reported results as *β* estimates with corresponding 95% confidence intervals (CIs) and as odds ratios (ORs) with 95% CIs. The OR reflects the change in AML risk per standard deviation (SD) increase in the genetically predicted exposure. A stepwise regression approach was adopted to screen potential exposures and mediators (Baron and Kenny 1986). We estimated the product of coefficients method (*β*_1_ × *β*_2_) as the indirect (mediated) effect, whereas the mediation proportion was estimated as (*β*_1_ × *β*_2_)/*β*.

## 3 Results

### 3.1 Genetic Instruments for Exposures

A total of 750 instrumental variables were identified for the 20 dietary factors, with a median *F*‐statistic of 46.11 (range: 29.7–646.73). From the GWAS data encompassing 412 gut microbiota categories, 4046 SNPs were selected, exhibiting a median *F*‐statistic of 21.64 (range: 19.51–61.10). Similarly, for 233 host metabolites, 13,507 SNPs were extracted, with a median *F*‐statistic of 133.53 (range: 29.72–7609.96). For AML used as the exposure, 14 SNPs were identified, with a median *F‐*statistic of 21.38 (range: 19.75–23.08). All selected SNPs had *F*‐statistics exceeding 10, ensuring robustness against weak instrument bias (Tables S3–S6).

### 3.2 Genetic Causal Effects of Dietary Factors on AML

**FIGURE 2:**
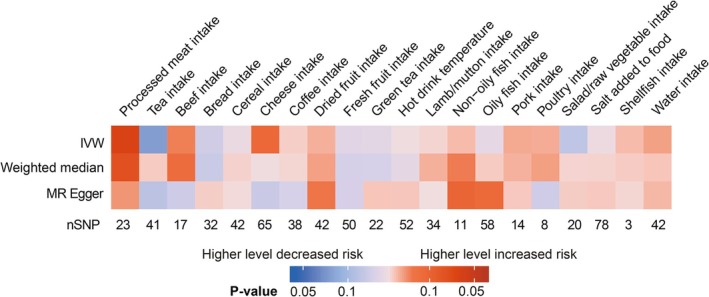
Mendelian randomization analyses reveal the causal influence of dietary factors on AML. The color gradient reflects the direction and strength of associations, with red indicating increased AML risk and blue indicating decreased AML risk. Darker shades signify stronger statistical significance (*P* < 0.05).

A two‐sample MR analysis using the IVW method initially identified associations between AML and 20 dietary factors across five categories: meat, vegetables, fruits, beverages, and seasonings. However, as shown in Figure 2, only processed meat intake remained statistically significant (IVW OR = 15.111; 95% CI = 1.253–182.262; *p* = 0.033) (Table S7), indicating a positive correlation with AML risk. Other MR methods yielded no statistically significant results (Figure 2B). Sensitivity analyses detected no evidence of horizontal pleiotropy or heterogeneity in the causal association between processed meat intake and AML (*P*_Q_ = 0.953, *P*_MR‐PRESSO_ = 0.955), reinforcing the robustness of the findings (Table S8).

To assess the stability of these results, a leave‐one‐out sensitivity analysis was performed, confirming that the association remained consistent regardless of SNP exclusion (Figure S1). Furthermore, reverse causality analysis provided no indication of a bidirectional relationship between processed meat intake and AML, further supporting the reliability of the observed causal effect (Table S9).

### 3.3 Genetic Causal Effects of Dietary Factors on Mediators

**FIGURE 3:**
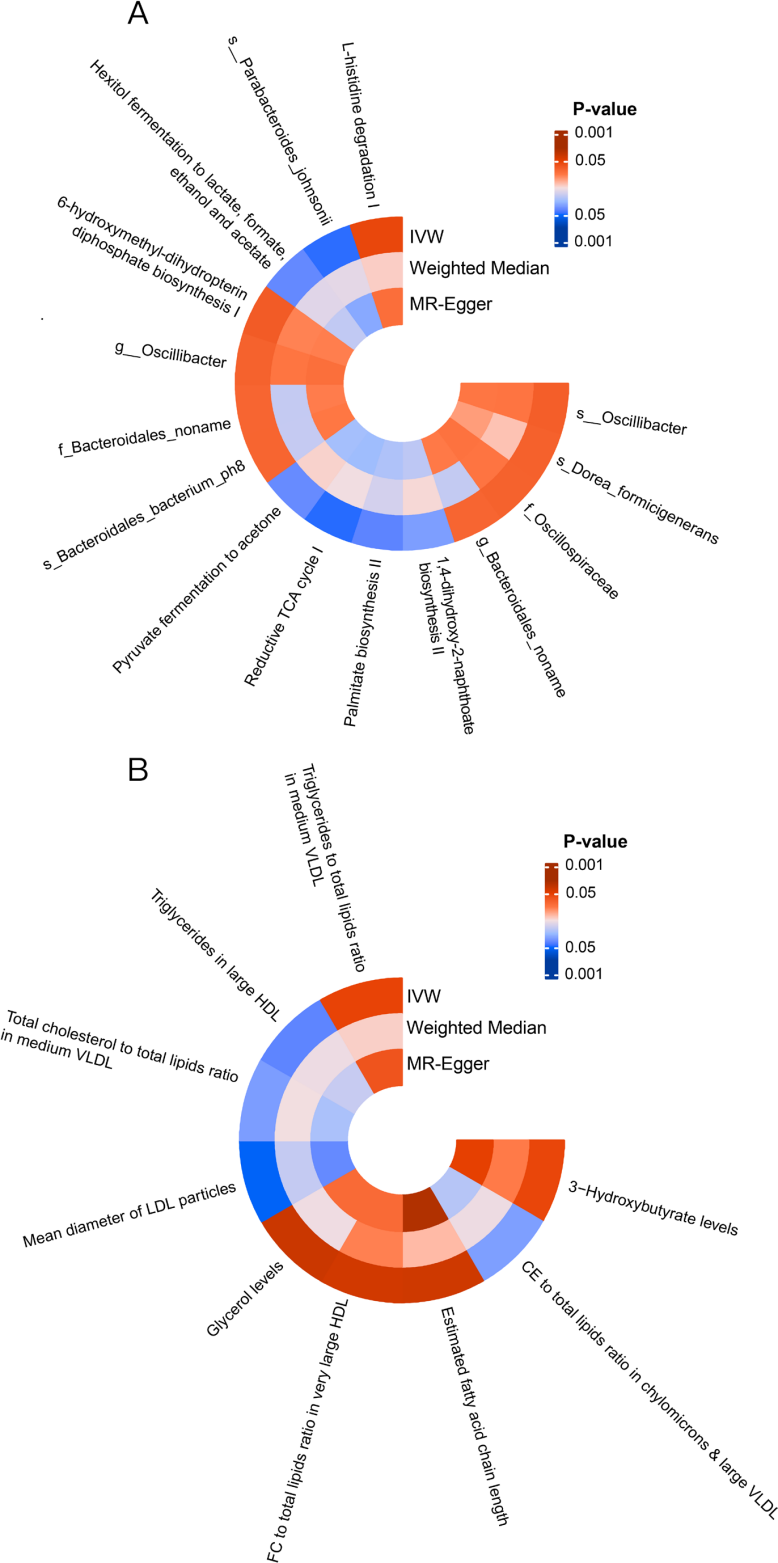
Mendelian randomization analyses reveal the causal influence of dietary factors on potential mediators. (A) Heatmap of gut microbiota categories as mediators. (B) Heatmap of host metabolites as mediators.

Our initial findings indicated a positive causal relationship between dietary factors and AML. To gain deeper insights into the underlying mechanisms, we conducted a two‐step MR analysis to assess potential mediating effects. In the first step, we examined the causal association between processed meat intake and potential mediators, including gut microbiota and host metabolites (Figure 3A). For gut microbiota and its associated metabolic pathways as mediators, 15 causally linked gut microbiota‐related categories were identified, encompassing both gut microbial taxa and metabolic pathways. These include seven gut bacterial metabolic pathways, namely L‐histidine degradation I pathway (IVW OR = 1.965; 95% CI = 1.152–3.352; *p* = 0.013), reductive TCA cycle I pathway (IVW OR = 0.224; 95% CI = 0.070–0.716; *p* = 0.012), hexitol fermentation to lactate, formate, ethanol, and acetate pathway (IVW OR = 0.522; 95% CI = 0.294–0.928; *p* = 0.027), 1,4‐dihydroxy‐2‐naphthoate biosynthesis II pathway (IVW OR = 0.465; 95% CI = 0.218–0.988; *p* = 0.047), Palmitate Biosynthesis II Pathway (IVW OR = 0.494; 95% CI = 0.268–0.909; *p* = 0.024), 6‐Hydroxymethyl‐Dihydropterin Diphosphate Biosynthesis I Pathway (IVW OR = 1.942; 95% CI = 1.067–3.535; *p* = 0.030), Pyruvate Fermentation to Acetone Pathway (IVW OR = 0.411; 95% CI = 0.185–0.910; *p* = 0.028). Moreover, eight gut microbiota were identified as potential mediating effects, such as Family‐level unclassified *Bacteroidales* (IVW OR = 1.754; 95% CI = 1.010–3.045; *p* = 0.046), Family *Oscillospiraceae* (IVW OR = 1.998; 95% CI = 1.033–3.865; *p* = 0.040), Genus‐level unclassified *Bacteroidales* (IVW OR = 1.754; 95% CI = 1.010–3.045; *p* = 0.046), Genus *Oscillibacter* (IVW OR = 1.997; 95% CI = 1.033–3.858; *p* = 0.040), *Bacteroidales bacterium ph 8* (IVW OR = 1.758; 95% CI = 1.013–3.053; *p* = 0.045), *Parabacteroides johnsonii* (IVW OR = 0.212; 95% CI = 0.063–0.713; *p* = 0.012), Species‐level unclassified *Oscillibacter* (IVW OR = 2.022; 95% CI = 1.057–3.868; *p* = 0.033), Species‐level *Dorea formicigenerans* (IVW OR = 2.274; 95% CI = 1.046–4.945; *p* = 0.038) (Table S10). Additionally, all tests passed checks for heterogeneity and pleiotropy (Table S11).

For host metabolites as mediators, we observed 9 categories have causal links (Figure 3B), namely 3‐hydroxybutyrate levels (IVW OR = 1.276; 95% CI = 1.052–1.527; *p* = 0.013), Cholesteryl Ester (CE) to total lipids ratio in chylomicrons & large VLDL (IVW OR = 0.854; 95% CI = 0.731–0.998; *p* = 0.048), Estimated fatty acid chain length (IVW OR = 1.342; 95% CI = 1.096–1.642; *p* = 0.044), Free cholesterol (FC) to total lipids ratio in very large high‐density lipoprotein (HDL) (IVW OR = 1.227; 95% CI = 1.064–1.415; *p* = 0.049), glycerol levels (IVW OR = 1.425; 95% CI = 1.121–1.811; *p* = 0.038), mean diameter of LDL particles (IVW OR = 1.843; 95% CI = 0.742–0.957; *p* = 0.008), total cholesterol‐to‐total lipids ratio in medium VLDL (IVW OR = 0.849; 95% CI = 0.723–0.996; *p* = 0.045), triglycerides in large HDL (IVW OR = 0.862; 95% CI = 0.758–0.981; *p* = 0.024), triglycerides to total lipids ratio in medium VLDL (IVW OR = 1.233; 95% CI = 1.048–1.451; *p* = 0.012) (Table S12). Moreover, two categories contain total cholesterol‐to‐total lipids ratio in medium VLDL (*P*_MR‐PRESSO_ = 0.027) and triglycerides to total lipids ratio in medium VLDL (*P*_MR‐PRESSO_ = 0.021) detected significant pleiotropy, while other tests passed checks for heterogeneity and pleiotropy (Table S13).

### 3.4 Genetic Causal Effects of Mediators on AML

**FIGURE 4:**
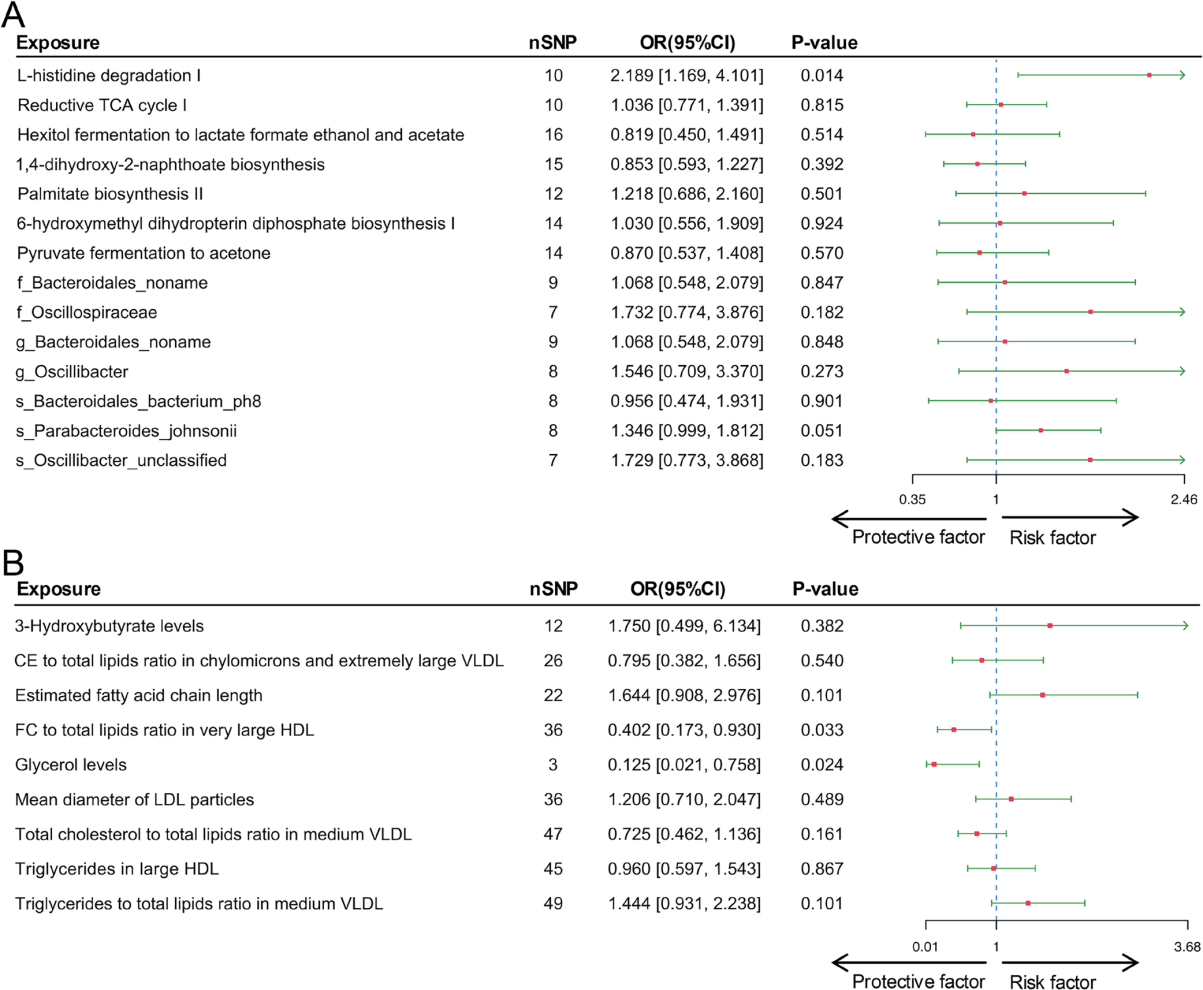
Mendelian randomization analyses reveal the causal influence of potential mediators on AML. (A) The exposure consists of positively associated gut microbiota categories identified with processed meat intake as the defining variable. (B) The exposure consists of positively associated host metabolites identified with processed meat intake as the defining variable.

To further substantiate the findings, a two‐sample MR analysis was employed to assess the causal roles of gut microbiota and host metabolites in AML. As a result, one gut bacterial pathway and two host metabolites were identified as having a causal relationship with AML (Figure 4). Genetically predicted higher activity of the L‐histidine degradation I pathway (IVW OR = 2.189; 95% CI = 1.169–4.101; *p* = 0.014) was associated with increased AML risk (Table S14), while higher FC to total lipids ratio in very large HDL (IVW OR = 0.402; 95% CI = 0.173–0.930; *p* = 0.014) and glycerol levels (IVW OR = 0.124; 95% CI = 0.021–0.758; *p* = 0.024) were associated with reduced risk (Table S15). Although this study employed a widely accepted lenient threshold (*p* < 1 × 10^−5^), the species‐level *Dorea formicigenerans* in the analysis still failed to include enough SNPs for IVW estimation. No significant pleiotropy and heterogeneity were detected among these gut microbiota and host metabolites (Tables S16 and S17).

### 3.5 Mediation Analysis

**FIGURE 5:**
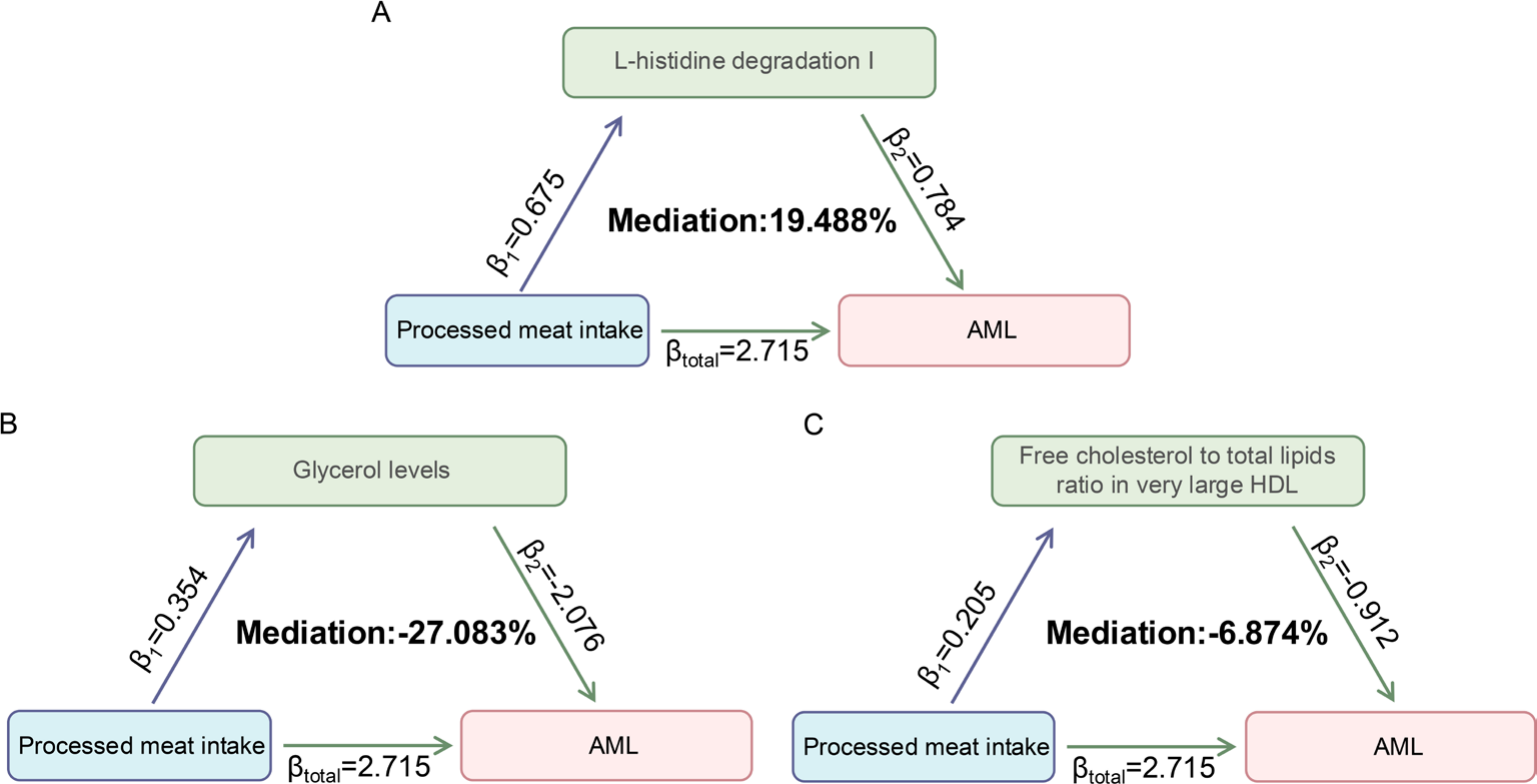
Positive mediators of the associations between processed meat intake and AML. (A) L‐histidine degradation I pathway mediates the association. (B) Glycerol levels mediate the association. (C) FC to total lipids ratio in very large HDL mediates the association.

Building upon the previous analysis, we identified three key biomarkers mediating the causal link between processed meat intake and AML. The total effect (*β* = 2.715) remained positive. Among the identified mediators, the L‐histidine degradation I pathway accounted for 19.488% of the total effect, highlighting its role as a gut microbiota‐associated positive mediator (Figure 5). In contrast, glycerol levels (−27.083%) and the FC to total lipids ratio in very large HDL (−6.874%) were found to exhibit protective mediating effects. This study employed a leave‐one‐out sensitivity analysis to assess whether the positive mediating effect of processed meat on AML causality was influenced by any single SNP. The results indicated that this mediating effect remained robust, regardless of the exclusion of any individual SNP (Figures S2–S5).

## 4 Discussion

This study conducted a comprehensive two‐sample, two‐step MR analysis to assess the causal relationship between 20 routine dietary factors and AML, with particular emphasis on the potential mediating roles of gut microbiota and host metabolites. These results suggest that processed meat intake may contribute to the development of AML, with evidence of mixed mediation effects involving both tumor‐promoting pathways and compensatory metabolic responses. Among the tumor‐promoting mediators, L‐histidine degradation I pathway, classified as gut microbiota‐mediated metabolic route, accounts for nearly 20% of the carcinogenic effect. In contrast, compensatory mediators such as host‐derived glycerol levels and the FC to total lipids ratio in very large HDL particles contribute approximately 27% and 7% of the protective effect, respectively. These observations may reflect an underlying self‐protective physiological response elicited by processed meat consumption. Notably, all identified mediators demonstrated robustness in sensitivity analyses, with no evidence of horizontal pleiotropy or between‐instrument heterogeneity.

Processed meat refers to meat products that have been preserved or flavored through methods such as salting, smoking, curing, or other similar methods. These processes generate known or suspected carcinogens (Bouvard et al. 2015). Specifically, heterocyclic aromatic amines (HAAs), along with curing agents such as potassium nitrate and sodium nitrite—which are added during meat processing—have been implicated as key carcinogenic contributors, particularly due to their ability to form N‐nitroso compounds (NOCs) under physiological conditions (Chazelas et al. 2022; Dong et al. 2020). With the accelerating pace of modern life and shifting dietary habits, the consumption of processed meat has markedly increased. Epidemiological studies have consistently reported a significant correlation between processed meat consumption and an elevated risk of breast cancer, colorectal cancer, colon cancer, rectal cancer, and lung cancer (Farvid et al. 2021). However, few studies have investigated the association between processed meat consumption and AML. A preliminary study reported a positive association between hot dog intake and the risk of childhood leukemia (Peters et al. 1994). Furthermore, a recent five‐year Japanese cohort study, which analyzed a combined cohort of 67 AML and 49 myelodysplastic syndrome (MDS) cases, reported that processed red meat consumption is a significant risk factor for AML/MDS (Shimomura et al. 2023). Nonetheless, prior studies are limited by the absence of RCT designs and a lack of mechanistic investigations elucidating how dietary factors contribute to the pathogenesis of AML. To address these limitations, the present study employs an MR approach, which leverages genetic variants as instrumental variables to infer causal relationships between processed meat intake and the risk of AML, thereby approximating the design of an RCT. Our analysis revealed a causal association between processed red meat consumption and the risk of AML, with an OR exceeding 10, indicating a potentially strong etiological contribution. These findings support the hypothesis that carcinogenic compounds present in processed meat may play a role in AML development, although the exact biological mechanisms remain to be elucidated.

Gut microbiota regulates intestinal mucosal barrier function primarily through the production of butyrate, a short‐chain fatty acid with anti‐inflammatory and barrier‐stabilizing properties. Moreover, alterations in gut microbiota composition have been closely linked to changes in muscle strength, redox homeostasis, and anorexia symptoms in patients with AML (Pötgens et al. 2024; Wang et al. 2022). Epidemiological evidence suggests that processed meat consumption can significantly alter the composition of the gut microbiota, potentially impacting host health through changes in microbial diversity and metabolic function (Maukonen et al. 2024). For instance, carcinogenic compounds commonly found in processed meat, such as NOCs, nitrite, and N‐nitrosodimethylamine (NDMA) have been associated with a decreased abundance of *Bifidobacterium bifidum* , suggesting that gut microbiota may serve as a key mediator influencing cancer susceptibility (Ruiz‐Saavedra et al. 2023). To date, few studies have explored the mediating role of the gut microbiota in the causal relationship between dietary factors and AML.

It is worth mentioning that processed meat consumption has been linked to the activation of the L‐histidine degradation I pathway in the gut microbiota, which may, in turn, contribute to an increased risk of AML. Lack of prognostic information limits our ability to perform a comprehensive analysis of the underlying associative mechanisms. However, we hypothesize that these mechanisms may involve the following pathways. First, the digestion of processed meat alters gut microbial metabolism, leading to an increased availability of L‐histidine substrates. This, in turn, stimulates the L‐histidine degradation I pathway in gut bacteria. Meanwhile, excessive histidine degradation depletes host L‐histidine levels, leading to histidine deficiency, which has been associated with enhanced leukemia cell survival and reduced sensitivity to chemotherapy. For instance, dietary histidine supplementation has been shown to enhance methotrexate sensitivity in leukemia cells by increasing flux through the histidine degradation pathway (Kanarek et al. 2018). Since L‐histidine serves as a precursor for histamine synthesis, histamine biosynthesis is consequently impaired when histidine availability is reduced. Studies have shown that histamine deficiency impairs myeloid cell maturation, leading to an accumulation of CD11b^+^Ly6G^+^ immature myeloid cells (IMCs), which are recruited during early carcinogenesis (Yang et al. 2011). In summary, our findings suggest that excessive consumption of processed meat activates the L‐histidine degradation pathway in the gut microbiota, resulting in systemic L‐histidine deficiency and, ultimately, an increased risk of AML. Targeting gut microbiota‐mediated histidine metabolism may therefore represent a novel therapeutic strategy for AML prevention and treatment. Further fundamental experiments and large‐cohort clinical studies are warranted to validate this hypothesis.

This study identified host metabolites that may mitigate the pathogenic effects of processed meat consumption on AML. Among these mediators, glycerol and the FC to total lipids ratio in very large HDL were found to exert protective roles. Glycerol, a key metabolic product of triglyceride hydrolysis, is released into circulation during lipolysis of dietary fats. Although glucose is typically the primary glycolytic substrate in cancer cells, glycerol can also be phosphorylated to form glycerol‐3‐phosphate, thereby feeding into both the glycolytic pathway and the tricarboxylic acid (TCA) cycle (Ganapathy‐Kanniappan and Geschwind 2013). However, the impact of increased glycerol levels in the circulatory system on cancer development remains unclear. Some studies suggest that exogenous glycerol supplementation can induce oxidative stress in cancer, subsequently activating p53 expression and promoting apoptosis in vivo (Capiglioni et al. 2018). Notably, AML cells exhibit higher metabolic demands than normal myeloid cells, making them potentially more sensitive to fluctuations in glycerol levels. Previous studies have shown that isocitrate dehydrogenase (IDH)‐mutant AML cells upregulate glycolysis via 2‐hydroxyglutarate (2‐HG), potentially increasing glycerol uptake to meet heightened bioenergetic and biosynthetic requirements (Savino and Stuani 2024). However, whether excessive glycerol uptake induces oxidative stress in AML cells and subsequently leads to AML cell death remains to be elucidated. Additionally, HDL levels are significantly higher in healthy individuals compared to AML patients and tend to increase following complete remission, suggesting a protective role for HDL particles in AML (Sincan et al. 2023). However, the specific subclasses of HDL and their protective roles in AML have not been fully characterized. Our research results suggested that FC to total lipids in very large HDL plays a protective role in AML, indicating that specific HDL subclasses may serve as potential protective factors in the progression of AML. Further investigation is warranted to address this knowledge gap and to clarify the functional roles of HDL subpopulations in leukemogenesis.

This study possesses several strengths. First, we leveraged the most recent and comprehensive GWAS datasets, ensuring that no sample overlap occurred between exposure and outcome datasets. Second, we employed rigorous instrumental variable selection, eliminating weak instrument bias, and performed systematic sensitivity analyses, enhancing the reliability and robustness of our findings. Third, the study elucidates key mechanistic pathways underlying the diet–gut microbiota–AML and diet–host metabolite–AML axes, thereby addressing limitations in previous studies that focused solely on single mediators. Furthermore, we identified causal pathways linking dietary exposures to gut microbiota and host metabolites, which could be leveraged for early diagnosis and the development of novel therapeutic strategies (Huang et al. 2024). Nonetheless, this study has several limitations. First, it was restricted to individuals of European populations, which may limit the generalizability of the findings to other ethnic populations. Second, the analysis focused solely on dietary patterns, without quantifying specific nutrient intake or accounting for variations in food processing methods. Third, the dietary exposure data were derived from UK Biobank participants aged 40–69 years, potentially limiting the applicability of these findings to younger or older individuals. Lastly, while our MR approach provided robust evidence for causal relationships, further large‐scale prospective clinical studies are required to validate these findings in real‐world settings.

## 5 Conclusions

This study offers new insights into the causal relationship between dietary factors and AML using a two‐step MR analysis. Our findings indicate that processed meat consumption significantly increases AML risk, with the gut microbiota‐associated L‐histidine degradation I pathway serving as a positive mediator. In contrast, the host metabolites glycerol and the FC to total lipids ratio in very large HDL may exert compensatory protective effects. These results underscore the complex and multifaceted interplay between diet, gut microbiota, and host metabolism in AML pathogenesis. Beyond advancing our mechanistic understanding, the identified microbial and metabolic mediators may serve as potential biomarkers for early detection or dietary‐based interventions. Future research should aim to replicate these findings across diverse populations and further elucidate the mechanistic roles of microbial and metabolic mediators in leukemogenesis.

## Author Contributions

**Jiading Qin:** formal analysis (equal), investigation (equal), methodology (equal), validation (equal), visualization (equal), writing – original draft (equal), writing – review and editing (equal). **Ling Zhang:** investigation (equal), methodology (equal), validation (equal). **Guangcheng Zhang:** investigation (equal). **Weijie Liao:** conceptualization (equal), resources (equal), supervision (equal), writing – review and editing (equal). **Li Yu:** conceptualization (equal), funding acquisition (lead), project administration (lead), supervision (lead), writing – review and editing (equal).

## Ethics Statement

Our study utilizes large‐scale GWAS datasets rather than individual‐level data. Ethical approval for the studies included in these consortia was granted by local research ethics committees and institutional review boards, with all participants providing written informed consent.

## Consent

The authors have nothing to report.

## Conflicts of Interest

The authors declare no conflicts of interest.

## Supporting information


**Figure S1.** MR leave‐one out sensitivity analysis for processed meat intake on AML.
**Figure S2.** MR leave‐one out sensitivity analysis for processed meat intake on gut bacterial pathway abundance (L‐histidine degradation I pathway).
**Figure S3.** MR leave‐one out sensitivity analysis for processed meat intake on circulating metabolites.
**Figure S4.** MR leave‐one out sensitivity analysis for gut bacterial pathway abundance (L‐histidine degradation I pathway) on AML.
**Figure S5.** Sensitivity analysis of the causal associations of positive mediating factors on AML.

**Table S1.** STROBE‐MR checklist of recommended items to address in reports of Mendelian randomization studies.
**Table S2.** Detailed information of questionnaire contents for 20 dietary habbits.
**Table S3.** Characteristics of dietary patterns genetic instrumental variables at the genome‐wide significance level.
**Table S4.** Characteristics of 412 gut microbiota genetic instrumental variables (1e‐05).
**Table S5.** Characteristics of 233 circulating metabolites genetic instrumental variables at the genome‐wide significance level.
**Table S6.** Characteristics of AML genetic instrumental variables (1e‐05).
**Table S7.** Detailed results of MR analysis for causal effects of dietary factors on AML.
**Table S8.** Detailed results of sensitivity analysis for causal effects of dietary factors on AML.
**Table S9.** Detailed results of reverse MR analysis for causal effects of AML on dietary factors.
**Table S10.** Detailed results of MR analysis for causal effects of dietary factors on gut microbiota as potential mediators.
**Table S11.** Detailed results of sensitivity analysis for causal effects of dietary factors on gut microbiota as potential mediators.
**Table S12.** Detailed results of MR analysis for causal effects of dietary factors on circulating metabolites as potential mediators.
**Table S13.** Detailed results of sensitivity analysis for causal effects of dietary factors on circulating metabolites as potential mediators.
**Table S14.** Detailed results of MR analysis for causal effects of gut microbiota on AML.
**Table S15.** Detailed results of sensitivity analysis for causal effects of gut microbiota on AML.
**Table S16.** Detailed results of MR analysis for causal effects of circulating metabolites on AML.
**Table S17.** Detailed results of sensitivity analysis for causal effects of circulating metabolites on AML.

## Data Availability

The GWAS summary statistics for dietary exposures were obtained from the UK Biobank via the IEU Open GWAS project (https://gwas.mrcieu.ac.uk/). Gut microbiota data were sourced from the Dutch Microbiome Project, which includes detailed taxonomic and functional pathway classifications. Host metabolite data were retrieved from publicly available datasets comprising 233 circulating human metabolic biomarkers quantified by NMR spectroscopy. AML outcome data were obtained from the FinnGen R12 dataset, a large population‐based biorepository in Finland. All datasets used in this study are publicly accessible, and further details are provided in the methods section. Additional data or materials may be available from the corresponding author upon reasonable request.
