# Supplementary material for: Genetically Predicted Gut Microbiota and Host Metabolites Mediate the Causal Link Between Dietary Factors and Acute Myeloid Leukemia
Source: Food Sci Nutr. 2025 Jun 23;13(6):e70456. doi: 10.1002/fsn3.70456 (PMC12183468; doi:10.1002/fsn3.70456)
Supplement: Supplementary file 1 — Figure S1. MR leave‐one out sensitivity analysis for processed meat intake on AML. Figure S2. MR leave‐one out sensitivity analysis for processed meat intake on gut bacterial pathway abundance (L‐histidine degradation I pathway). Figure S3. MR leave‐one out sensitivity analysis for processed meat intake on circulating metabolites. Figure S4. MR leave‐one out sensitivity analysis for gut bacterial pathway abundance (L‐histidine degradation I pathway) on AML. Figure S5. Sensitivity analysis of the causal associations of positive mediating factors on AML. [file FSN3-13-e70456-s002.docx]

Figure S1. MR leave-one out sensitivity analysis for processed meat intake on AML


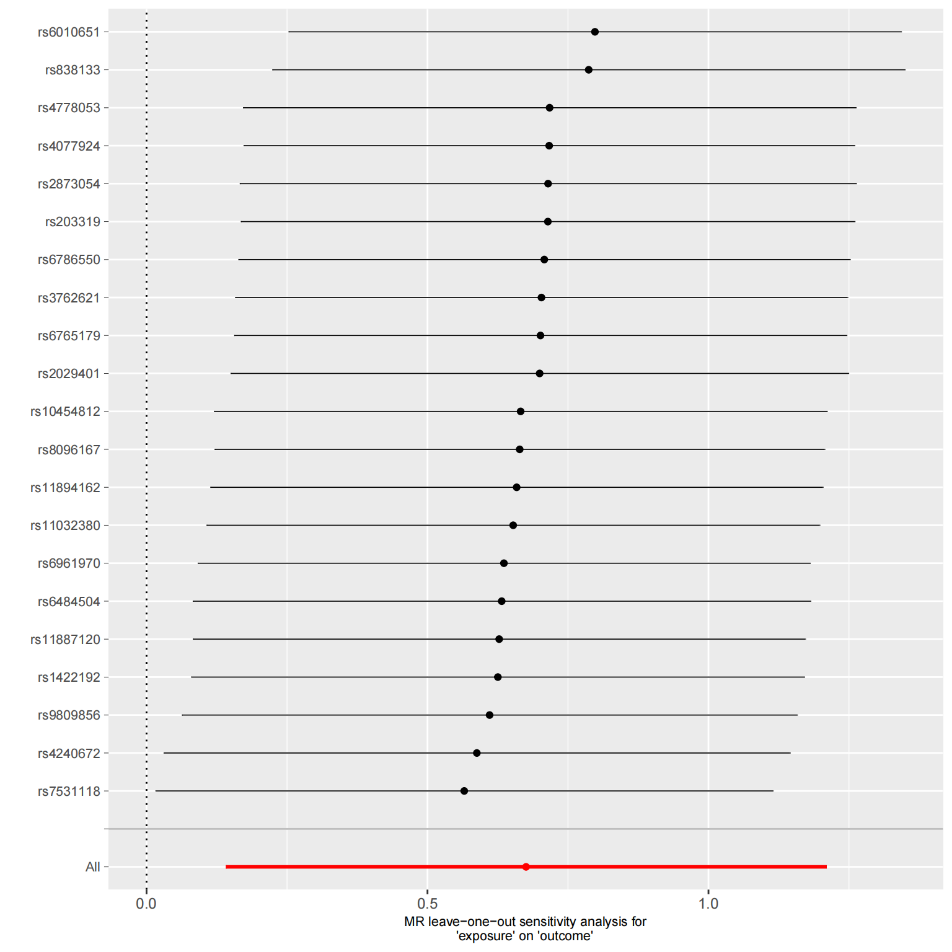
Figure S2. MR leave-one out sensitivity analysis for processed meat intake on Gut bacterial pathway abundance (L-histidine degradation I pathway)


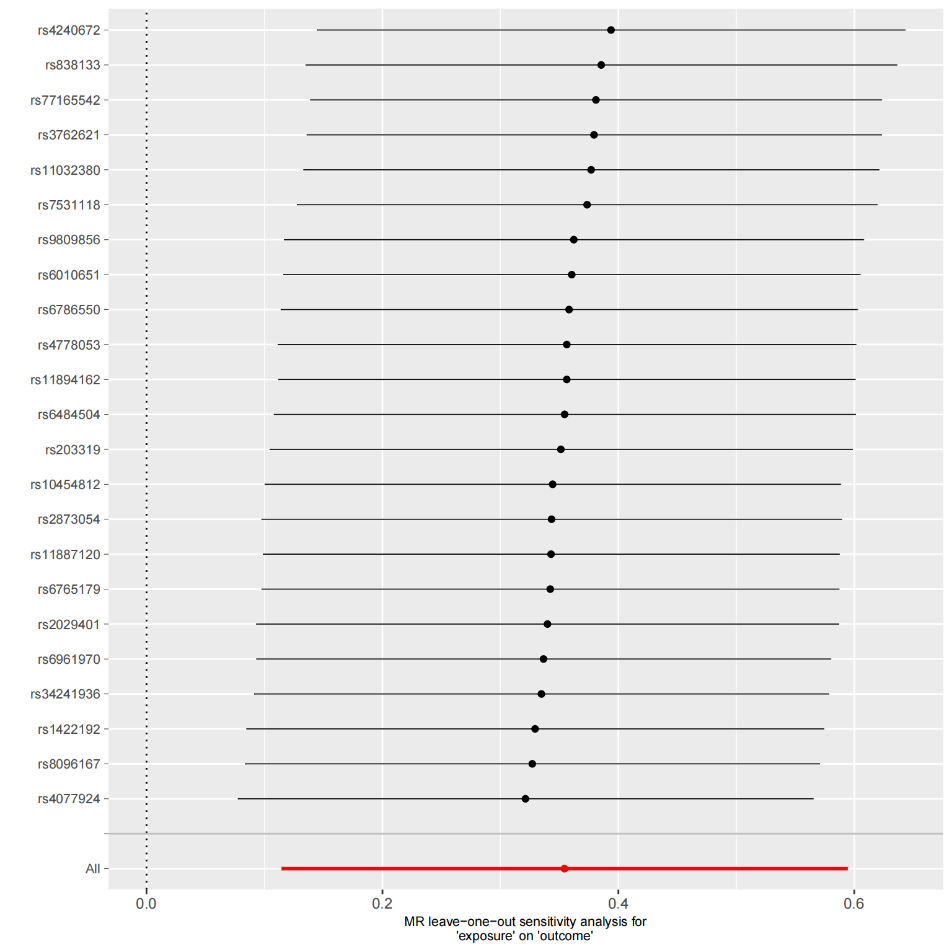

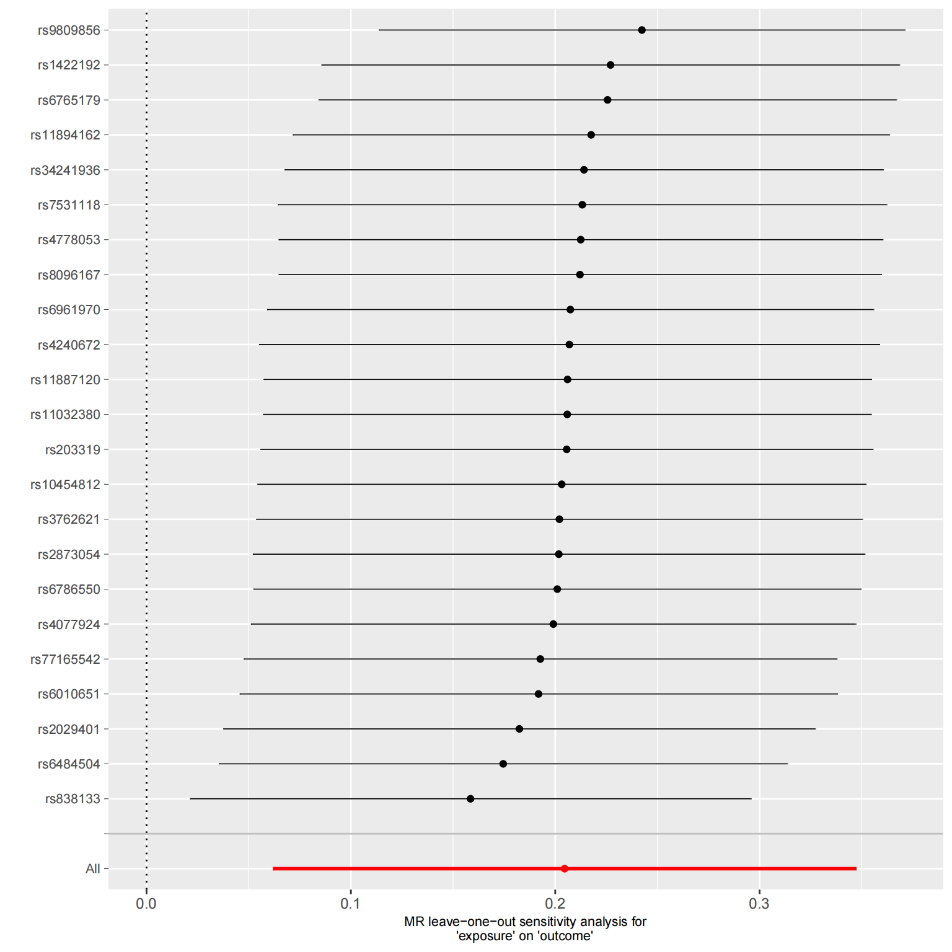
Figure S3. MR leave-one out sensitivity analysis for processed meat intake on circulating metabolites. (A) FC to total lipids ratio in very large HDL. (B) Glycerol levels

(B)

(B)

(A)


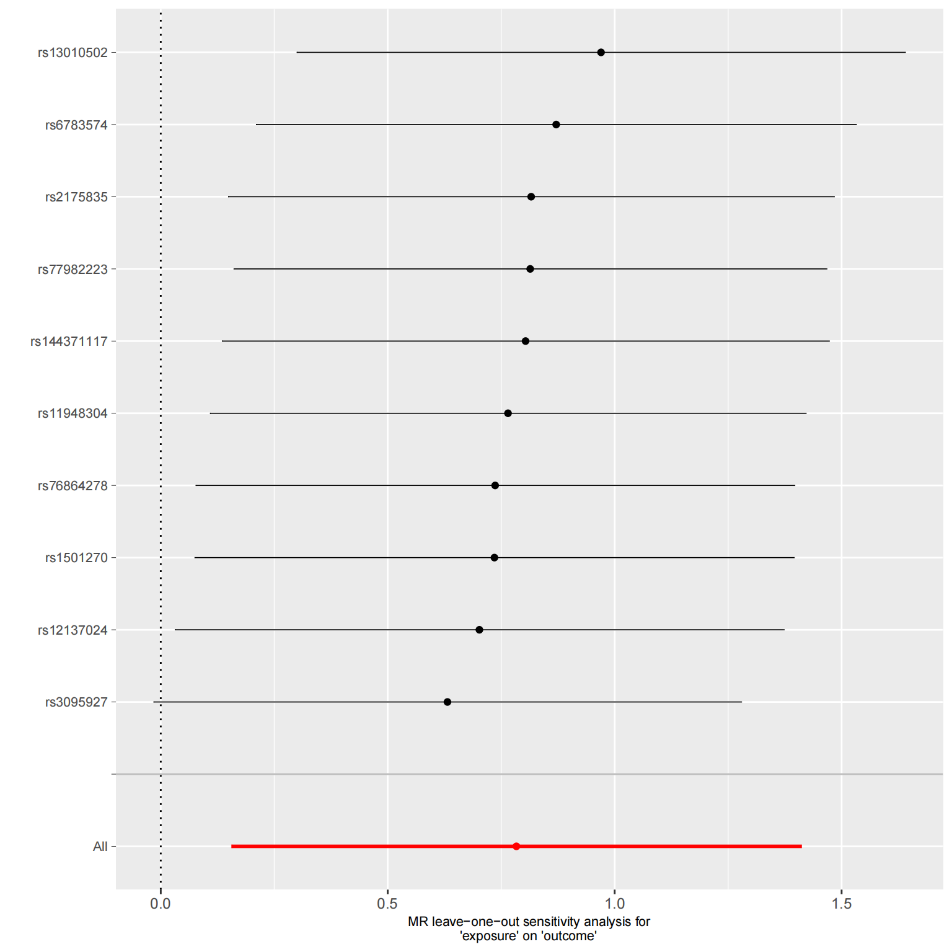
Figure S4. MR leave-one out sensitivity analysis for Gut bacterial pathway abundance (L-histidine degradation I pathway) on AML


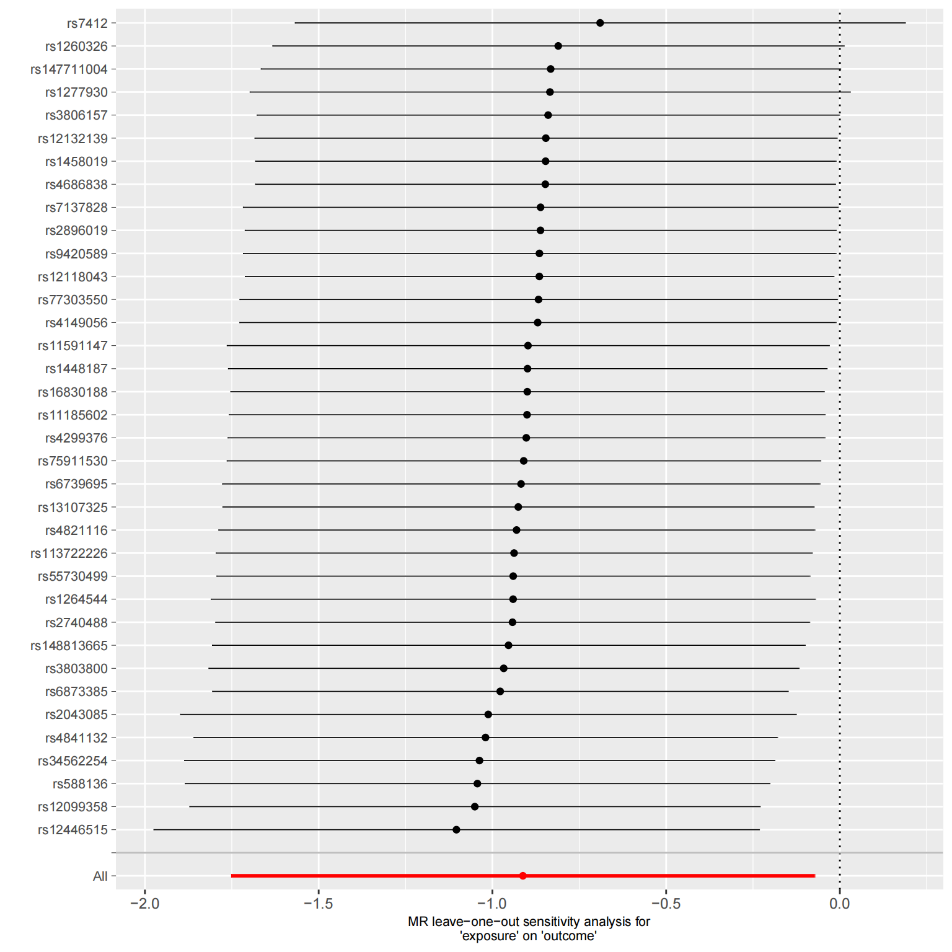

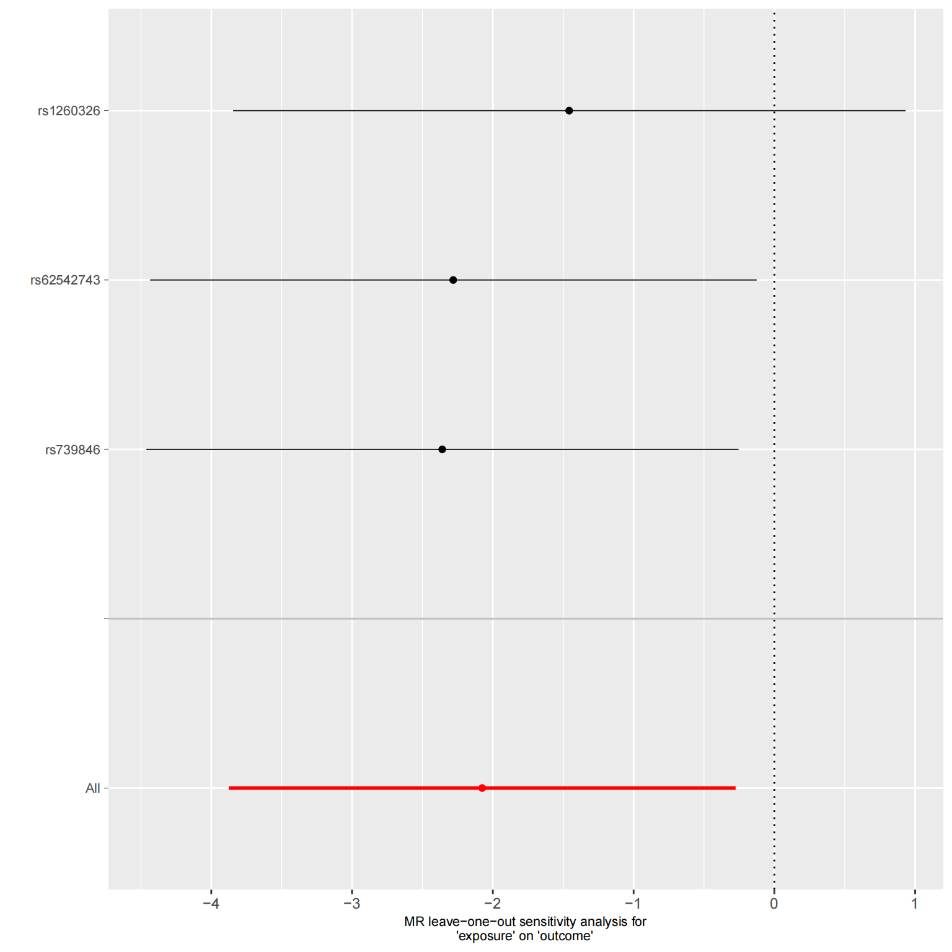
Figure S5. Sensitivity analysis of the causal associations of positive mediating factors on AML. (A) Glycerol levels. (B) Free cholesterol to total lipids ratio in very large HDL

(A)

(B)
